# Cre-Dependent Expression of Multiple Transgenes in Isolated Neurons of the Adult Forebrain

**DOI:** 10.1371/journal.pone.0003059

**Published:** 2008-08-26

**Authors:** Sridhara Chakravarthy, Tara Keck, Martijn Roelandse, Robin Hartman, Andreas Jeromin, Sean Perry, Sonja B. Hofer, Thomas Mrsic-Flogel, Christiaan N. Levelt

**Affiliations:** 1 Molecular Visual Plasticity Group, Netherlands Institute for Neuroscience, Amsterdam, The Netherlands; 2 Department of Cellular and Systems Neurobiology, Max Planck Institute of Neurobiology, Martinsried, Germany; 3 Allen Institute for Brain Science, Seattle, Washington, United States of America; Minnesota State University Mankato, United States of America

## Abstract

**Background:**

Transgenic mice with mosaic, Golgi-staining-like expression of enhanced green fluorescent protein (EGFP) have been very useful in studying the dynamics of neuronal structure and function. In order to further investigate the molecular events regulating structural plasticity, it would be useful to express multiple proteins in the same sparse neurons, allowing co-expression of functional proteins or co-labeling of subcellular compartments with other fluorescent proteins. However, it has been difficult to obtain reproducible expression in the same subset of neurons for direct comparison of neurons expressing different functional proteins.

**Principal Findings:**

Here we describe a Cre-transgenic line that allows reproducible expression of transgenic proteins of choice in a small number of neurons of the adult cortex, hippocampus, striatum, olfactory bulb, subiculum, hypothalamus, superior colliculus and amygdala. We show that using these Cre-transgenic mice, multiple Cre-dependent transgenes can be expressed together in the same isolated neurons. We also describe a Cre-dependent transgenic line expressing a membrane associated EGFP (EGFP-F). Crossed with the Cre-transgenic line, EGFP-F expression starts in the adolescent forebrain, is present in dendrites, dendritic protrusions, axons and boutons and is strong enough for acute or chronic in vivo imaging.

**Significance:**

This triple transgenic approach will aid the morphological and functional characterization of neurons in various Cre-dependent transgenic mice.

## Introduction

The development and functionality of the central nervous system involves complex interactions between various neuronal cell types. Therefore, tools that allow us to visualize isolated neurons of a particular class are of immense help in the analysis of the dynamic connectivity during development and adulthood. For more than a century, the majority of morphological studies in neuroscience at the cellular and subcellular level have been carried out using the Golgi approach to stain individual neurons [1] and visualize their dendrites and dendritic protrusions. Although this approach has been vital for the elucidation of the structural details of the central nervous system, it is not suitable for examining the dynamic functions of neurons.

The advent of green fluorescent protein (GFP) as a tool to label cells [2] together with live cell imaging by confocal- [3]–[6] or two photon microscopy [7]–[10] have permitted the study of dynamic structural changes occurring with development and plasticity of the central nervous system in vivo [11]–[13]. Various methods have been developed to express GFP in sparse neurons in vitro and in vivo, necessary for studying neuronal morphology. Particle-mediated gene transfer allows labeling of individual neurons in slice cultures [14]. Viral-mediated gene delivery has proven ideal for mosaic gene expression in vivo [15], [16] but can also be used in slice cultures [17], [18].

Transgenic approaches, though more time consuming, have the advantage of being non-invasive and less variable. Transgenic mice expressing various spectral variants of GFP under the control of the Thy1 promoter, with distinct expression patterns in the cortex have been produced [19] and proved very useful for the analysis of the dynamics of synapse formation and pruning in vivo [20]–[22]. Unfortunately, only limited expression patterns are currently available, making it impossible to study synapse dynamics of other structures in the CNS including the supragranular layers of the visual cortex.

For studying molecular mechanisms regulating dynamics of morphological changes, similar transgenic mice with two additional features are necessary. The first is that the mosaic expression pattern can be reproduced with novel fluorescent- or functional transgenic proteins to allow direct comparison of their effects in the exact same subset of neurons. The second feature is that multiple transgenes can be expressed in the same individual neurons. This allows the labeling of neuronal morphology with GFP and the concurrent expression of fluorescent synaptic markers or functional transgenes enabling the analysis of cell autonomous processes and the dissociation of pre- and postsynaptic mechanisms.

The first feature has been achieved previously by making use of the Cre/Lox system to achieve mosaic transgene expression. The mosaic expression pattern is defined by the Cre-transgenic mice and crossing them to lines carrying different Cre-dependent transgenes allows their expression in a reproducible fashion. Only four such mice have been described until now. The first line shows expression in very low numbers of excitatory neurons in the neocortex in a columnar pattern [23]. As the localization of these columns is unpredictable, these mice are not very suitable for in vivo two photon imaging. The other lines express hormone-analogue inducible versions of the Cre recombinase. [24]–[26]. Though useful for addressing questions depending on precise timing of transgene expression, the main caveat of this approach is that concentrations of estrogen homologues required for activating Cre approach those that affect dendritic growth and spine morphology [27].

The second feature, expressing multiple transgenes in the same individual neurons, has been very difficult to achieve. Although it is theoretically possible to express two proteins in a Cre-dependent fashion using bicistronic vectors, this approach usually results in relatively low expression of one of the transgenic proteins in vivo. Recently, a novel technology named “mosaic analysis with double markers” (MADM) was introduced, which allows the mosaic expression of multiple fluorescent proteins and manipulation of endogenous genes at the same time [28]. Though a powerful approach, it is technically very demanding, involving the production of multiple gene-targeted animals for each gene of interest to be studied or fluorescent protein to be expressed.

To overcome these issues, we describe here a Cre-transgenic line that can mediate expression of multiple Cre-dependent transgenes of choice in very few neurons of the adolescent neocortex, hippocampus, olfactory bulb, striatum, amygdala, hypothalamus and superior colliculus. Moreover, a novel Cre-dependent GFP transgenic line is described allowing chronic imaging of synaptic structures when combined with the mosaic Cre-line.

## Results

### Generation of transgenic mice

**Figure 1:**
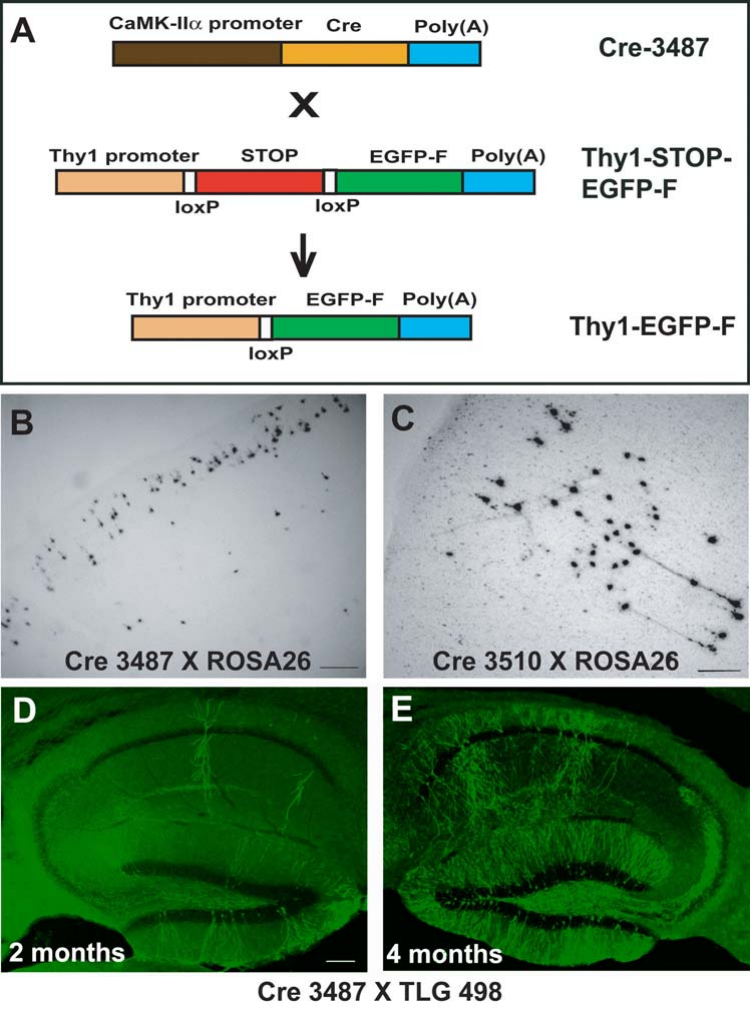
Generation of Cre-dependent mosaic transgenic mice. (A) Transgenic mice based on Cre/Lox recombination were generated by crossing a Cre-transgenic line under a CaMKIIα promoter with a Thy1 promoter-driven EGFP-F transgenic line containing a transcriptional STOP cassette. Bright field microscopy images showing mosaic expression of the β-galactosidase expressing cells in layers II/III and V/VI of the neocortex of R26R mice when crossed with different CaMKIIα promoter-driven Cre-transgenic lines – (B) Cre 3487 and (C) Cre 3510. Epi-fluorescence microscopy images of Cre-dependent EGFP-F expression in TLG 498^+^ Cre 3487^+^ transgenic mice. EGFP-F expression starts at 6 weeks and increases with age. The number of EGFP-F^+^ cells increases from (D) 2 months to (E) 4 months of age. Scale bars: B, D-100 µm; C-200 µm.

Transgenic mice were generated based on the Cre/Lox-recombination approach [29]. Using a construct encoding the Cre recombinase under the control of the Calcium/Calmodulin dependent kinase IIα (CaMKIIα) promoter (Fig. 1A) four founders were created. These lines defined the expression pattern and onset of reporter gene expression. Initially they were crossed with R26R mice that express the β-galactosidase gene under the endogenous ROSA26 promoter in a Cre-dependent fashion [30]. This allowed us to check for the efficiency of Cre recombination by analyzing the expression pattern of β-galactosidase expression. The offspring of two founder lines (Cre 3487 and 3510) showed Cre recombination in a small subset of cells (Fig. 1B and 1C). Line Cre 3510 showed columnar Cre recombination similarly to the mice described earlier. Line Cre 3487 showed a more even distribution of labeled neurons over the neocortex and hippocampus and was used in all subsequent experiments.

The second construct used for the production of transgenic animals mediated expression of EGFP-F under the control of the Thy1 promoter (Fig. 1A). A transcriptional STOP cassette flanked by two loxP sites was inserted between the promoter and coding region, ensuring that the timing and expression pattern of this transgene was dependent on the Cre-transgenic animals they were to be crossed to. Ten EGFP-F founder lines were created and crossed initially to a broad expressing Cre-transgenic line. One of the lines, TLG 498, showed good levels of EGFP-F expression, in more than 90% of pyramidal neurons in the neocortex and hippocampus. Another line, TLG 696, showed good expression levels of EGFP-F, but only in 60–70% of all pyramidal neurons. Line TLG 1157 expressed high levels of EGFP-F in all pyramidal neurons. Line TLG 706 showed extremely high levels of EGFP-F causing the protein to accumulate in the soma. Most quantitative data was obtained with line TLG 498 as it was the first line produced with good EGFP-F expression levels. Line TLG 1157 showing higher expression levels of EGFP-F was produced later and was used predominantly for in vitro and vivo imaging experiments.

### Cre-mediated transgenes are expressed in individual neurons of the adult brain

**Figure 2:**
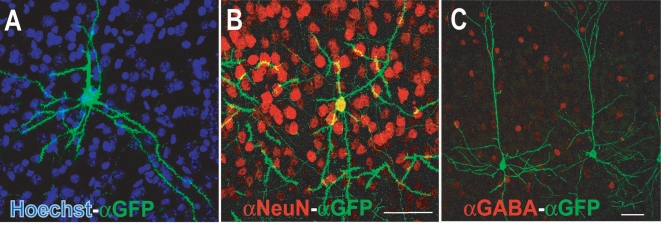
Mosaic expression of EGFP-F in the cortex is confined to excitatory neurons. (A) A confocal image of a 50 µm section of the visual cortex of two month old TLG 498^+^ Cre 3487^+^ transgenic mice for antibodies against GFP (green) and counterstained with Hoechst 33342 (blue nuclei) shows the low recombination efficiency. (B) Double immunofluorescence for antibodies against GFP (green) and the neuronal marker NeuN (red) show colocalization of NeuN in EGFP-F expressing cells. (C) In the cortex there is no colocalization of EGFP-F with GABA, a marker for inhibitory neurons (red). Scale bar: 50 µm.

**Figure 3:**
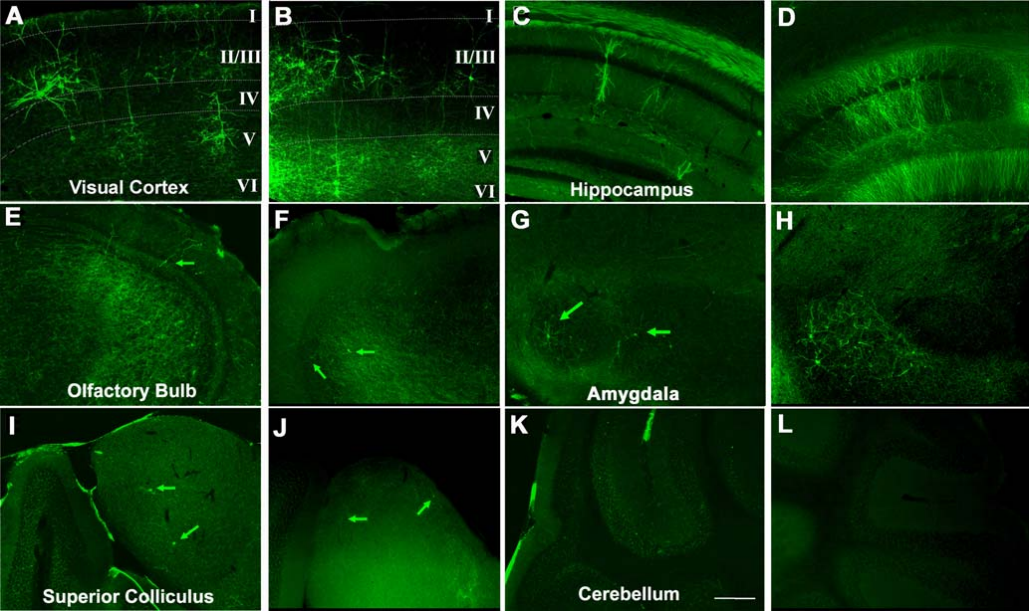
Mosaic transgene expression in various parts of the adult forebrain. Epi-fluorescence microscopy images showing expression of EGFP-F in TLG 498^+^ Cre 3487^+^ transgenic mice at 2 months of age in (A) pyramidal cells in layers II/III and V/VI of visual cortex, C) pyramidal and granule cells of hippocampus, (E) granule cells of the olfactory bulb, (G) pyramidal cells of amygdala and (I) piriform cells in superior colliculus (arrows). There is no expression in the cerebellum (K). Scale bar −200 µm. A very similar expression pattern was observed in 2½ month old TLG 1157^+^ Cre 3487^+^ transgenic mice as shown in the juxtaposed figures (same scale): (B) pyramidal cells in visual cortex, (D) granule cells of hippocampus, (F) granule cells of the olfactory bulb, (H) pyramidal cells of amygdala, and (J) piriform cells in superior colliculus. Again, no expression was observed in cerebellum (L).

**Figure 4:**
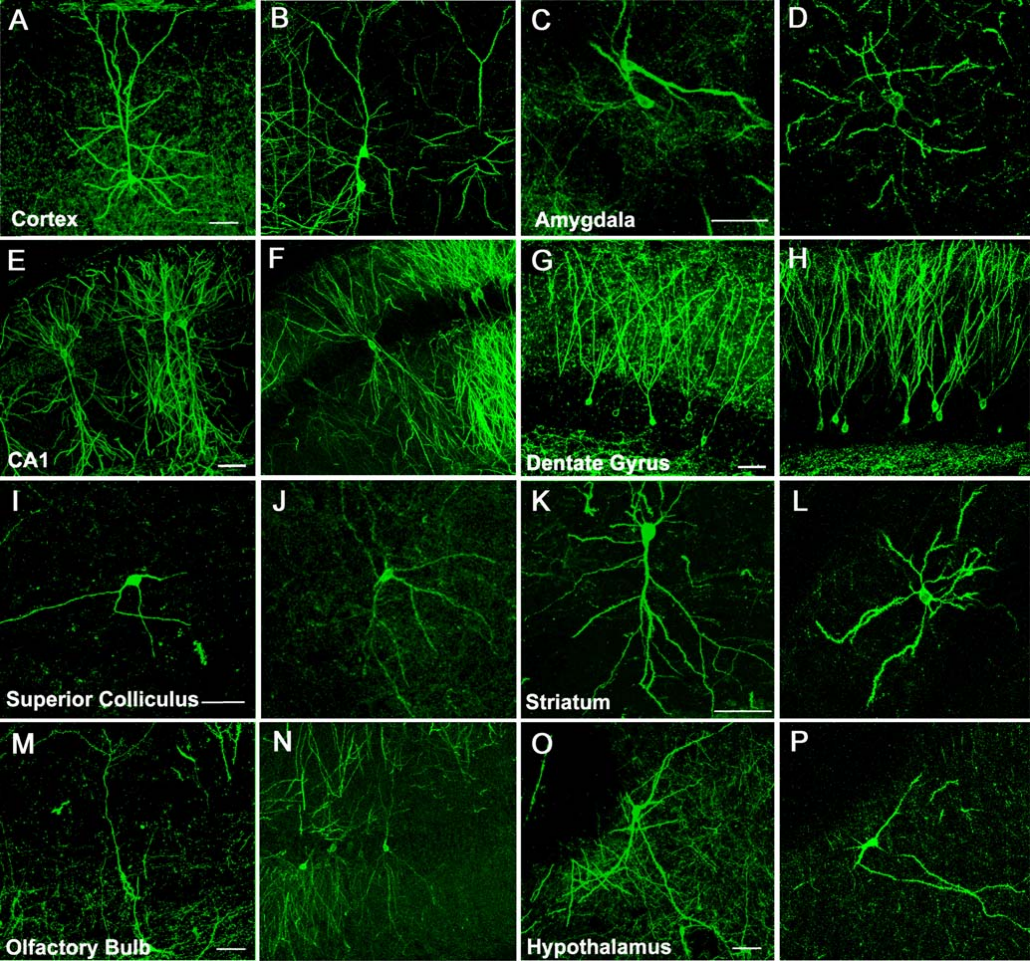
Cre-dependent EGFP-F labels distinct neuronal types in the adult forebrain. Confocal microscopy images showing that mosaic EGFP-F expression in a 2 month old TLG 498^+^ Cre 3487^+^ mouse occurs in pyramidal cells in (A) neocortex, (C) amygdala and (E) hippocampal CA1; (G) granule cells in the dentate gyrus; (I) marginal cells in the superior colliculus; (K) spiny projection neurons in the striatum; (M) granule cells in the olfactory bulb and (O) tubero-mammillary cells in the posterior hypothalamus. Scale bar −50 µm.Mosaic EGFP-F expression in a 2½ month old TLG 1157^+^ Cre 3487^+^ transgenic mouse occurs in the same cell types as shown by the juxtaposed figures (B, D, F, H, J, L, N, P – same scale as juxtaposed images).

In TLG 498^+^ Cre 3487^+^ double transgenic animals, mosaic EGFP-F expression started at around 5–6 weeks after birth and accumulated during the following weeks (Fig. 1 D–E). Immunohistochemical staining for the neuronal marker NeuN confirmed that transgene expression was confined to neurons (Fig. 2B). EGFP-F^+^ cells in the neocortex and hippocampus did not express GABA, a marker for inhibitory neurons (Fig. 2C). Both in TLG 498^+^ Cre 3487^+^ and TLG 1157^+^ Cre 3487^+^ double transgenic animals EGFP-F fluorescence was detected in pyramidal neurons predominantly in layer II/III, but also V and VI of the neocortex (Fig. 3A–B) and in pyramidal and granule cells of the hippocampus (Fig. 3C–D). Within the neocortex, EGFP-F expressing neurons were observed in the visual cortex, somatosensory cortex, auditory cortex, lateral orbital cortex, piriform cortex, agranular insular cortex and retrospenial agranular cortex. EGFP-F expression in individual cells was also observed in the olfactory bulb (Fig. 3E–F), subiculum, hypothalamus, striatum, amygdala (Fig. 3G–H) and superior colliculus (Fig. 3I–J). There was no expression in the thalamic nuclei or cerebellum (Fig. 3K–L). Counterstaining the sections with Hoechst 33342 allowed the determination of recombination efficiency. In the visual cortex, approximately one out of a thousand cells expressed EGFP-F (Fig. 2A) whereas more cells recombined in the agranular insular, cortex and hippocampus. In order to represent this low recombination efficiency in a more practically applicable manner, we counted the total number of EGFP-F^+^ cells from various regions of interest from 50 µm thick sections of TLG 498^+^ Cre 3487^+^ tanimals (Table 1). Morphological analyses in TLG 498^+^ Cre 3487^+^ and TLG 1157^+^ Cre 3487^+^ double positive animals identified EGFP-F^+^ neurons in the extragranular layers (II, III, V and VI) of the neocortex (Fig. 4A–B), amygdala (Fig. 4C–D), and in hippocampal CA1 (Fig. 4E–F) and CA3 as pyramidal cells, and as granule cells in dentate gyrus (Fig. 4G–H). In superior colliculus only marginal cells and piriform neurons expressed EGFP-F (Fig. 4I–J). Striatal EGFP-F^+^ expressing cells were medium spiny projection neurons (Fig. 4K–L), while in olfactory bulb, EGFP-F^+^ cells were granule cells (Fig. 4M–N). In the hypothalamus, EGFP-F was expressed by the tubero-mammillary neurons (Fig. 4O–P). These EGFP-F^+^ neurons conform to the subset of cell types which express high levels of endogenous CaMKIIα. Frequently, several EGFP-F expressing neurons were found in close proximity to each other.

**Table 1:** Recombination efficiencies in various areas of the adult brain.

Region	Cell Type	Number of EGFP^+^ cells
Motor Cortex	Pyramidal	16±4.7
Somatosensory Cortex	Pyramidal	7.6±2.0
Visual Cortex	Pyramidal	7.2±4.5
Retrosplenial Granular Cortex	Pyramidal	2.3±1.9
Subiculum	NV	1.8±1.0
Hippocampus – CA	Pyramidal	11±7.5
Hippocampus - Dentate Gyrus	Granule	13±4.5
Striatum	Medium spiny projection	3.4±2.2
Olfactory Bulb	Granule	29±7.8
Anterior Olfactory Nucleus	NV	6.3±2.7
Olfactory Tubercle	NV	10±2.8
Amygdala	Pyramidal	2.2±1.6
Hypothalamus	Tubero-mammillary	5.3±3.2
Superior Colliculus	Piriform and Marginal	2.1±1.6

EGFP-F^+^ cells were counted from various brain regions of four 2 month old TLG 498^+^ Cre 3487^+^ mice. A total of 17 sections of 50 µm thickness were used. The average number of EGFP-F expressing cells/section in each part of the forebrain is shown. Error bars represent standard deviation between sections. Cell types were identified by morphology alone. NV – not verified.

### Multiple transgenes can be expressed in the same neuronal population

For studying molecular mechanisms underlying structural plasticity, it is often required to express multiple transgenes in the same individual neurons. Therefore, we tested whether in TLG 498^+^ R26R^+^ Cre 3487^+^ triple transgenic mice, both EGFP-F and β-galactosidase would be expressed in the same individual neurons.

TLG 498^+^ R26R^+^ Cre 3487^+^ triple transgenic mice were generated by first producing mice double transgenic for the marker genes (TLG 498^+^ and R26R^+^). Next, these mice were crossed with Cre 3487 transgenic animals. The offspring that were TLG 498^+^ R26R^+^ Cre 3487^+^ were used to determine the percentage of EGFP-F^+^ cells that expressed β-galactosidase. We generally avoided using TLG 498^+^ Cre 3487^+^ transgenic mice for further breeding, as germline recombination sometimes occurred resulting in broad EGFP-F expression in the next generation. In case the breeding of mice carrying a floxed gene and Cre 3487^+^ is essential for some reason, we recommend genetic testing of the offspring to exclude germline recombination before use or further breeding.

**Figure 5:**
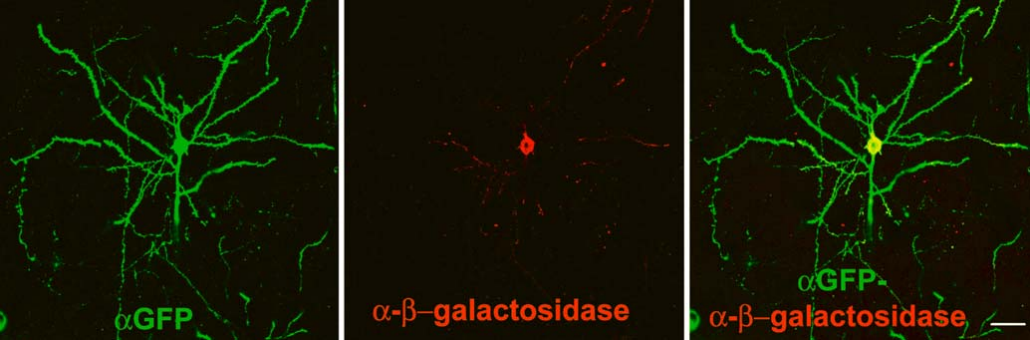
Multiple transgenes can be expressed in the same cell. As determined by confocal microscopy, the resulting R26R^+^ TLG 498^+^ Cre 3487^+^ offspring express both EGFP-F (green) and β-galactosidase (red) in a Cre-dependent fashion. β-Galactosidase is predominantly localized perinuclearly and partly in the dendrites whereas EGFP-F labels the entire neuron. Scale bar: B −25 µm.

TLG 498^+^ R26R^+^ Cre 3487^+^ offspring were used for cell counts. β-galactosidase was predominantly present close to the soma and partly in the proximal dendrites whereas EGFP-F was seen throughout the neuron (Fig. 5). Cell counts showed that 80–90% of EGFP-F^+^ cortical cells in layer II/III and V/VI also expressed β-galactosidase (Table 2) and a similar percentage of β-galactosidase^+^ neurons also expressed EGFP-F.

**Table 2:** Colocalization of EGFP-F and β-galactosidase in TLG 498^+^ R26R^+^ Cre 3487^+^ mice.

Cortical Layer	% Colocalization of β-galactosidase and EGFP-F^+^	
	EGFP-F^+^ cells expressing β-galactosidase	β-galactosidase^+^ expressing EGFP-F
Layer II/III	79.5% (120/151)	92.3% (120/130)
Layer V/VI	87% (33/38)	92% (33/35)

Two month old TLG 498^+^ R26R^+^ Cre 3487^+^ transgenic mice were analyzed for colocalization of β-galactosidase in EGFP-F^+^ cells of the visual cortex. A total of 8 sections of 100 µm thickness were used from 3 mice and 201 cells were counted. Numbers between brackets are absolute numbers of cells counted. Between 80–90% of neurons expressing β-galactosidase also express EGFP-F and vice versa.

### Visualization of neuronal structures of EGFP-F expressing neurons

**Figure 6:**
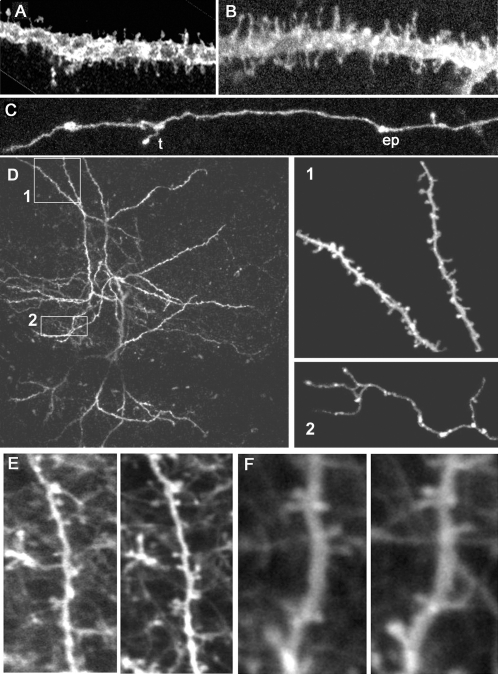
Membrane expression of EGFP-F labels dendritic spines and axonal boutons (A–C) Detailed confocal microscopy images show that the farnesylation site on EGFP-F targets its expression predominantly to the membrane surface in TLG 1157^+^ Cre 3487^+^ mice. EGFP-F labels large and small dendritic spines. (C) Terminaux (t) and en passant (ep) axonal boutons are clearly labeled by EGFP-F. (D) Z-projection of a stack acquired by in vivo two photon imaging of the visual cortex of a ten week old TLG 498^+^ Cre 3487^+^ transgenic animal. The collapsed image shows dendrites of two neighboring layer V pyramidal neurons, 75–100 µm from the pial surface. Dendritic protrusions (D1) and axonal boutons (D2) are clearly visible. (E–F) Z-projections of stacks acquired by chronic in vivo two photon imaging of the visual cortex of a 4 month old TLG 1157^+^ Cre 3487^+^ transgenic animal. Dendrites with dendritic spines of different pyramidal neurons imaged 7 and 9 days (E) and 12 and 15 days (F) after window implantation, 75–100 µm from the pial surface.

We next analyzed whether the subcellular localization of EGFP-F allowed the analysis of neuronal structures. The farnesylation modification of EGFP ensured it to be targeted to the membrane (Fig. 6A and B, line TLG 1157 shown). EGFP-F fluorescence was detected evenly distributed throughout the membrane of the cell including the dendritic spines (Fig. 6A–B) and axons (Fig. 6C). Small and thin spines, the stalks of large spines as well as filopodia could be clearly visualized. Both types of axonal boutons – en passant and terminaux – were labeled with EGFP-F (Fig. 6C). Subcellular distribution of EGFP in line TLG 498 was the same, albeit a lower expression levels [31]. Biocytin injections confirmed that all dendritic protrusions were labeled with EGFP-F [31]. We did notice that in both lines, cells expressed varying levels of EGFP-F, which may possibly depend on the number of copies of the transgene that are activated by Cre recombination.

### Strong expression of EGFP-F allows chronic in vivo imaging of neurons and dendritic protrusions

The advantage of using GFP variants to label neurons is the ability to follow dynamic changes in cellular and subcellular compartments. Here, we tested if the levels of EGFP-F expression in TLG 498^+^ Cre 3487^+^ and TLG 1157^+^ Cre 3487^+^ double transgenic mice were sufficient for *in vivo* two photon imaging. In TLG 498^+^ Cre 3487^+^ double transgenic animals, only the brightest EGFP-F expressing cells in layers II/III, V or VI could be imaged with in vivo two-photon microscopy and dendritic protrusions, axons and their boutons were visible in such cells (Fig. 6D). EGFP-F expressing neurons were, however, often not bright enough for repeated imaging in TLG 498^+^ Cre 3487^+^ mice (data not shown). In contrast, EGFP-F expressing cells could be imaged without difficulty in TLG 1157^+^ Cre 3487^+^ mice and EGFP-F levels were more than sufficient for repeated imaging (Fig 6E and F).

## Discussion

In vivo two–photon imaging of transgenic mice with mosaic, Golgi-staining like GFP expression has made the analysis of the dynamics of structural changes in neurons possible. This approach has resulted in the elucidation of its basic properties including the stability and turnover of dendritic spines of neurons from various cortical regions during early developmental and in adult mice [20]–[22]. Importantly, it has provided insight into the effects of sensory experience and neuronal activity on the motility and stability of dendritic spines and filopodia [13], [32]–[34]. However, many fundamental questions still remain unanswered. For example, it is still unknown how changes in the precise connectivity between different subsets of neurons mediate the functional changes that are achieved, which molecular and cellular processes regulate these events and why increased plasticity can take place during sensitive periods. Here we describe Cre- and membrane associated EGFP (EGFP-F)-transgenic lines allowing the co-expression of multiple independent transgenes in the same isolated neurons in various brain areas starting 5–6 weeks after birth. This late onset of Cre-recombination allows studying the involvement of specific proteins in the adult CNS as unwanted effects of transgene expression during development are avoided. The mice will be a welcome new tool for studying molecular events regulating synapse dynamics in the adult brain as proteins of choice can be expressed in a reproducible manner, allowing direct comparison of neurons expressing multiple fluorescent and/or functional proteins.

In line Cre-3487, expression of Cre is driven by the CamKIIα promoter and results in mosaic activation of Cre-dependent reporter genes in brain areas and cell types known to express high levels of endogenous CamKIIα. These cell types include pyramidal neurons of layers II/III and V/VI of the neocortex, pyramidal cells of CA1 and CA3, and granule cells of the dentate gyrus. Recombination also occured in pyramidal neurons in amygdala; marginal and piriform cells in the superior colliculus; medium spiny projection neurons in striatum, granule cells in the olfactory bulb and tubero-mammillary neurons in the hypothalamus.

We initially believed that the mosaicism mediated by the CamKII-Cre transgene was caused by low – rather than mosaic – expression of the Cre recombinase, translating into infrequent (stochastic) activation of Cre-dependent transgenes. We reasoned that in that case the likelihood that multiple Cre-dependent transgenes would be activated in the same individual neurons is very small. However, after crossing in two Cre-dependent transgenes (encoding EGFP-F and β-galactosidase respectively) we observed that 80–90% of cortical neurons expressing one transgene also expressed the other, suggesting that Cre-expression itself is mosaic. Moreover, considering the high level of co-expression of these independent Cre-dependent transgenes and our observation that two different Cre-dependent EGFP-F transgenic lines showed transgene expression in the same sparse neuronal subsets when crossed to the Cre-3487 line, we conclude that the mosaic expression pattern observed is caused by the Cre-3487 line and not by the reporter lines.

Mosaic expression of fluorescent proteins in the CNS is essential for studying dynamic changes of synaptic connectivity using live imaging techniques. In our new Cre-dependent EGFP-F line, TLG 1157, EGFP-F expression in pyramidal neurons of layers II/III, V and VI was sufficient to permit acute and chronic in vivo imaging of neurites and synaptic structures when crossed to Cre-3487 transgenic mice. These double transgenic mice will be an attractive new addition to the extensively used mosaic GFP transgenic lines produced by Feng et al. [19], especially because none of these lines show extensive GFP expression in layer II/III neurons in the primary visual cortex. We anticipate that the Cre- and EGFP-F transgenic animals described here will also be useful for studying molecular mechanisms regulating neuronal function and plasticity. As multiple transgenes can be expressed in the same isolated neurons using the Cre-3487 transgenic animals, it should be possible to express other transgenes together with EGFP-F from line TLG 1157. Such transgenes may encode fusion proteins with red fluorescent proteins labeling subcellular structures of choice allowing the analysis of synapse formation, vesicle transport, cytoskeletal rearrangements or other processes with live cell imaging techniques if sufficiently high expression levels are accomplished. Alternatively, proteins altering specific signaling pathways can be expressed. In this case it would be advisable to co-express a red fluorescent protein using an internal ribosomal entry site in order to allow monitoring for co-expression of both transgenes as this will not occur in all neurons. Considering that by making use of the Thy1-promoter in combination with a floxed stop cassette, one out of ten EGFP-F transgenic founders had high enough expression for chronic imaging of small structures and two more had sufficient expression for identification of transgene expressing neurons, this approach seems highly feasible. Of course, Cre-dependent reporter lines other than the EGFP-F lines described here can be utilized in combination with line Cre-3487. Especially using the recently described Brainbow reporter mice [36] provides interesting options as it will allow studying the effects of specific signaling pathways on synaptic connectivity in exquisite detail.

## Materials and Methods

### DNA constructs and production of transgenic mice

The construct for expressing the EGFP-F in neurons in a Cre-dependent fashion was created as described previously [31]. In short: the lox-Stop-lox (LSL) cassette from PBS302 (Gibco-BRL, Bethesda, MD) was cloned into a Thy1 promoter containing expression vector, rendering pThy-LSL. pThyLSL-EGFP-F was created by cloning the fragment encoding EGFP fused to the H-Ras farnesylation site from pEGFP-F (BD Biosciences, Mountain View, CA) into pThy-LSL. For production of transgenic mice expressing Cre under the control of the CaMKIIα promoter, pJTCre [37] was employed (Fig. 1A). Transgenic mice were created by pronuclear injections of linearized DNA into fertilized C57BL/6 oocytes. All mice analyzed were in kept in or backcrossed to C57BL/6 background for at least 5 generations. To initially analyze the recombination efficiency in new Cre-transgenic founders, we crossed them to R26R reporter mice. All experiments involving mice were approved by the institutional animal care and use committee of the Royal Netherlands Academy of Arts and Sciences. Cre- and transgenic lines can be obtained from the transgenic unit of the Netherlands Institute for Neuroscience by contacting the corresponding author.

### Histology and immunohistochemistry

Mice of various ages (4–20 weeks) were anaesthetized with 0.1 ml/gr bodyweight Nembutal (Janssen Pharmaceutical, UK) and transcardially perfused with 4% paraformaldehyde (PFA) in PBS. Triple transgenic mice were perfused at 8 weeks. Brains were post-fixed for 2 hrs and stored at 4°C in PBS. Coronal sections of 50 µm were made using a vibratome (Leica VT1000S, Leica, Rijswijk, Netherlands). Immunohistochemistry was performed on free-floating sections using mouse anti-GFP antibodies (1∶500, Chemicon) followed by Alexa 568 conjugated goat anti-mouse IgG antibodies (1∶500, Molecular Probes). Subsequent to the incubation with secondary antibodies, sections were counterstained with 5 µg/ml Hoechst 33342 (Sigma) for 5 minutes and then washed three times with PBS containing 0.1% Triton.

Double immunohistochemistry for EGFP-F and additional histochemical markers was performed using chicken anti-GFP antibodies (1∶1000, Chemicon) followed by Alexa488 conjugated anti-chicken IgG antibodies (1∶500, Molecular Probes) and rabbit anti-GABA (1∶100, Medical Performance Products, The Netherlands) followed by Alexa 568 conjugated anti-rabbit IgG antibodies (1∶500, Molecular Probes) or mouse anti-NeuN (1∶100, Chemicon) followed by Alexa 568 conjugated anti-mouse IgG antibodies (1∶500, Molecular Probes). Double immunohistochemistry for EGFP-F and β−galactosidase were performed with biotin-labeled mouse anti-β−galactosidase antibodies (1∶1000; Sigma) followed by Cy3 conjugated streptavidin (1∶500, Vector Laboratories) along with chicken anti-GFP antibodies followed by Alexa 488 conjugated secondary antibody. For enzymatic β-galactosidase detection, brain sections from the R26R mice crossed with Cre transgenic animals were incubated for 1 hour at 37°C with X-Gal-solution containing 5 mM K_4_Fe(CN)_6_, 5 mM K_3_Fe(CN)_6_, 2 mM MgCl_2_, 1 mg/ml 5-bromo4-chloro-3-indolyl-D-galactoside (Invitrogen, Carlsbad, CA) in PBS. The X-Gal reacted sections were washed with PBS, dehydrated and embedded in entellan mounting medium.

### Microscopy, image analysis and cell counts

Stained or unstained EGFP-F expressing neurons were imaged using a Leica DRM microscope (Leica-Microsystems, Bensheim, Germany) for epifluorescence images, or using a Carl Zeiss CLSM 510 Meta confocal microscope (Zeiss, Goettingen, Germany) with Argon (488 nm) and HeNe (543 nm) lasers. Hoechst 33342 stained sections were imaged with a tunable Ti∶Sapphire laser (Mai Tai, Spectraphysics, Darmstadt, Germany) running at 725 nm. Confocal images were made using a 20× objective (0.5 NA) with 1 µm steps in the z-plane. Spines and axons were imaged at a pixel resolution of 60 nm (63× oil objective, 1.4 NA, and an optical zoom of 2.5×) with 200 nm steps in the z-plane. The back-projected pinhole was 190 nm. For each image acquisition of whole neurons, the laser intensity and detector gain were adjusted so that the entire detector range was used for the dendrites. When imaging at a higher magnification, the detector range was set for the spines and axons.

Recombination efficiency was determined by counting the number of EGFP-F^+^ cells on sections that were stained with anti-GFP antibodies. Different brain regions where identified by making use of the Paxinos mouse brain atlas and the number of EGFP-F expressing neurons in that region where counted.A total of 17 sections of 50 µm thickness were used from four 2 month old mice.

### In vivo two-photon imaging

Two to four month old mice were imaged using in vivo two-photon microscopy. The mice were anesthetized and maintained with intraperitoneal injections of Fentanyl: (0.05 mg/kg), Midazolam: (5.0 mg/kg), Medetomidin: (0.5 mg/kg). The depth of anaesthesia was tested by foot-pinch reflex. A homeostatic heating blanket was used to maintain the body temperature at 37°C and the heart rate was monitored by placing the front and hind paws of the mouse on electrodes with hydrogel. Atropine and dexamethasone were given subcutaneously to the mice before surgery. The skull was removed overlying the visual cortex using a motorized drill. The dural surface was covered by a thin layer of 1.2% agarose and a coverslip (5 mm diameter), whose edges were subsequently sealed by dental acrylic. A thin titanium head bar was attached next to the cranial window. Neurons were imaged with a custom-built microscope and a mode-locked Ti∶sapphire laser (Mai Tai, Spectra-Physics) at 910 nm through a 40× water immersion objective (0.8 NA, Olympus). Scanning and image acquisition were controlled by Fluoview software (Olympus). The average power delivered to the brain was <50 mW. Images stacks (1024×1024 pixels, in 0.5 um steps) of different magnifications were taken within the upper 100 µm from the pial surface. In acute experiments, mice were imaged immediately following surgery. In chronic experiments, the animals were imaged starting one week after cranial window implantation. Imaging occurred at 2–4 day intervals.
